# The resuscitation-promoting factor (Rpf) from *Micrococcus luteus* and its putative reaction product 1,6-anhydro-MurNAc increase culturability of environmental bacteria

**DOI:** 10.1099/acmi.0.000647.v4

**Published:** 2023-09-22

**Authors:** Juan Guzman, Dipansi Raval, Dirk Hauck, Alexander Titz, Anja Poehlein, Thomas Degenkolb, Rolf Daniel, Andreas Vilcinskas

**Affiliations:** ^1^​ Department of Bioresources, Fraunhofer Institute for Molecular Biology and Applied Ecology, Giessen, Germany; ^2^​ Institute for Insect Biotechnology, Justus-Liebig-University of Giessen, Giessen, Germany; ^3^​ Chemical Biology of Carbohydrates, Helmholtz Institute for Pharmaceutical Research Saarland (HIPS) – Helmholtz Centre for Infection Research (HZI), Saarbrücken, Germany; ^4^​ German Center for Infection Research, site Hannover-Braunschweig, Saarbrücken, Germany; ^5^​ Department of Chemistry, Saarland University, Saarbrücken, Germany; ^6^​ Genomic and Applied Microbiology and Göttingen Genomics Laboratory, Institute of Microbiology and Genetics, University of Göttingen, Göttingen, Germany

**Keywords:** resuscitation-promoting factors, *Micrococcus luteus*, *Actinobacteria*, cultivation

## Abstract

Dormant bacterial cells do not divide and are not immediately culturable, but they persist in a state of low metabolic activity, a physiological state having clinical relevance, for instance in latent tuberculosis. Resuscitation-promoting factors (Rpfs) are proteins that act as signalling molecules mediating growth and replication. In this study we aimed to test the effect of Rpfs from *Micrococcus luteus* on the number and diversity of cultured bacteria using insect and soil samples, and to examine if the increase in culturability could be reproduced with the putative reaction product of Rpf, 1,6-anhydro-*N*-acetylmuramic acid (1,6-anhydro-MurNAc). The *rpf* gene from *Micrococcus luteus* was amplified and cloned into a pET21b expression vector and the protein was expressed in *Escherichia coli* BL21(DE3) cells and purified by affinity chromatography using a hexa-histidine tag. 1,6-Anhydro-MurNAc was prepared using reported chemical synthesis methods. Recombinant Rpf protein or 1,6-anhydro-MurNAc were added to R2A cultivation media, and their effect on the culturability of bacteria from eight environmental samples including four cockroach guts and four soils was examined. Colony-forming units, 16S rRNA gene copies and Illumina amplicon sequencing of the 16S rRNA gene were measured for all eight samples subjected to three different treatments: Rpf, 1,6-anhydro-MurNAc or blank control. Both Rpf and 1,6-anhydro-MurNAc increased the number of colony-forming units and of 16S rRNA gene copies across the samples although the protein was more effective. The Rpf and 1,6-anhydro-MurNAc promoted the cultivation of a diverse set of bacteria and in particular certain clades of the phyla *Actinomycetota* and *Bacillota *. This study opens the path for improved cultivation strategies aiming to isolate and study yet undescribed living bacterial organisms.

## Data Summary

- *
Streptomyces* sp. strain G173LV was deposited in culture collections as DSM 112694 and CCM 9166, respectively.
- *
Gordonia* sp. strain G177LV was deposited in culture collections as DSM 112678 and CCM 9158.
- The 16S rRNA gene of *Streptomyces* sp. strain G173LV was deposited in GenBank with accession number OP753711.
- The 16S rRNA gene of *Gordonia* sp. strain G177LV was deposited in GenBank with accession number MZ182279.
- The 16S rRNA gene amplicon sequencing raw reads were deposited in the NCBI Sequence Read Archive with accession number BioProject PRJNA968073.

## Introduction

Resuscitation-promoting factors (Rpfs) are secreted bacterial proteins responsible for stimulating the growth of sibling cells, when these have entered a dormant state induced by nutritional deprivation or additional environmental stresses [1]. Dormant cells do not divide and are thus not immediately culturable, but they persist in a reversible state of low metabolic activity. Dormancy or cellular quiescence has been traditionally observed in non-sporulating *Actinobacteria *, such as *Mycobacterium tuberculosis *, while sporulating bacteria evolved a more extreme form of dormancy in which spores shut down all metabolic processes [2]. Notably, Rpfs are encoded by both sporulating and non-sporulating *Actinobacteria* [3, 4], suggesting an ancestral mechanism of awakening. The first Rpf was purified from the supernatant of a *Micrococcus luteus* culture, and found to be highly active, with picomolar concentrations causing a 100-fold increase in the number of dividing viable cells [5]. The Rpf protein from *Micrococcus luteus* resuscitates other actinobacteria including rhodococci and mycobacteria [6]. Reciprocally, the Rpfs from *Mycobacterium tuberculosis* resuscitated *Micrococcus luteus* cells [7]. The genomes of both *Mycobacterium tuberculosis* and *Streptomyces coelicolor* encode five Rpfs, but they have different domain organization [8]. Rpfs display host specificity, being particularly efficient in resuscitating closely related taxa and probably evolved as messengers to awake sibling cells [9].

Rpf proteins share structural homology to lysozyme [10], and they display muralytic activity [11, 12]. However, unlike lysozyme, Rpfs cleave the β-1,4 glycosidic bond between *N*-acetyl-glucosamine (GlcNAc) and *N*-acetyl-muramic acid (MurNAc) while simultaneously forming 1,6-anhydro derivatives of muramic acid [13]. The release of small hydrolytic Rpf products from the peptidoglycan stimulates resuscitation [14]. Specifically *N*-acetylglucosaminyl-β(1→4)-*N*-glycolyl-1,6-anhydromuramyl-l-alanyl-d-isoglutamate was found to resuscitate *Mycobacterium smegmatis* [15].

The addition of Rpf to isolation media has been used to improve the recovery of bacteria from environmental samples, resulting in the cultivation of novel species of *Actinobacteria* [16–19]. Moreover, Rpf enhances biodegradation of noxious contaminants by environmental bacteria [20, 21]. The supernatant of *Micrococcus luteus* increased the abundance of cultured bacteria, and particularly of the phylum *Pseudomonadota *, during cultivation of soil samples [22]. In the present study, we measured the increase in the number and diversity of cultured bacteria by addition of recombinant *Micrococcus luteus* Rpf. We also examined the effect of adding 1,6-anhydro-*N*-acetyl-muramic acid (1,6-anhydro-MurNAc), one of the simplest potential lytic products from the action of Rpf on the peptidoglycan.

## Methods

### Chemicals and samples

1,6-Anhydro-MurNAc was prepared synthetically from GlyNAc as described previously [23].

The cockroaches *Archimandrita tesselata* (AT), *Blaberus craniifer* (BC), *Gromphadorhina portentosa* (GP) and *Lucihormetica verrucosa* (LV) were purchased in 2018 and 2019 from Jörg Bernhardt (see http://www.schaben-spinnen.de/). The insects were identified using molecular fingerprinting by sequencing the mitochondrial cytochrome *c* oxidase subunit I (COI) as reported previously [24]. The cockroaches were cleaned with water, and the guts were carefully dissected and suspended in 500 µl of 50 % (v/v) sterile glycerol and thoroughly cut into very fine fragments using sterile surgical scissors. Four different soils (soils1 and 4 were black, and soils2 and 3 were sandy soils) were collected in a plastic bag using a spade in the areas near the Fraunhofer IME institute in Giessen in March 2022. The soil samples (500 mg) were mixed with 500 µl of 50 % (v/v) sterile glycerol. The suspensions were vortexed at full speed for 1 min, and then centrifuged at 2432 ***g*** for 10 s. The supernatant (200 µl) was transferred into a clean and sterile 1.5 ml tube and used for downstream analysis.

### Cloning of the *rpf* gene and recombinant Rpf expression

DNA from *Micrococcus luteus* strain DSM 20030^T^ was extracted from a 10 ml overnight culture in casein soy peptone (CASO) broth, using a modified Marmur purification method [25]. Both insert (*rpf* gene from *Micrococcus luteus *) and backbone pET21b(+) were amplified with the respective primers MlrpfF/R and pET21bF/R (Table 1) using Q5 High-Fidelity DNA Polymerase (NEB) as recommended by the manufacturer in a 50 µl reaction volume. The PCR temperature programme used was 98 °C for 30 s, followed by 35 cycles of 98 °C for 10 s, 50 °C for 30 s and 72 °C for 30 s, and final elongation at 72 °C for 2 min. After subsequent separation by agarose gel electrophoresis, both fragments were excised and purified using a GeneJET Gel Extraction Kit (Thermo Scientific). The *Micrococcus luteus-*rpf insert (749 bp) and the pET21b backbone (5 369 bp) have a 20 bp overlap introduced with the primers and were assembled using NEBuilder HiFi reagent (NEB). The fragments were mixed in a 5 : 1 backbone/insert ratio and incubated with the reagent for 30 min at 50 °C. We used 3 µl of the unpurified assembled reaction mixture to transform 50 µl of 5-alpha competent *Escherichia coli* cells (NEB). The reaction was incubated with 450 µl SOC medium for 1 h at 37 °C and 250 r.p.m. shaking. Thereafter, an aliquot of 100 µl was plated on LB agar containing 100 mg l^−1^ ampicillin. The next day, a colony was selected and cultured overnight in 10 ml LB broth with 100 mg l^−1^ ampicillin. The plasmid was purified using a PureLink quick plasmid miniprep (Invitrogen) from 1.5 ml of overnight culture and eluted with 75 µl of TE buffer. The plasmid was checked for correct in-frame insert positioning by amplification with primers T7promF and T7termR (Table 1), followed by agarose gel electrophoresis, amplicon purification and Sanger sequencing at GATC Eurofins. The confirmed plasmid pET21bMlutrpf was used to transform 50 µl of BL21(DE3) competent *E. coli* cells (NEB). A colony was selected and cultured in 200 ml LB broth with 100 mg l^−1^ ampicillin, at 37 °C and 180 r.p.m. shaking. When the optical density at 600 nm reached 0.6, the cells were induced with 1 mM IPTG and further incubated at 18 °C for 16 h. The culture was centrifuged at 2 500 ***g*** for 15 min, and the pellet was resuspended in lysis buffer containing 20 mM Tris/HCl, 100 mM NaCl, 10 % glycerol and 10 mM imidazole at pH 8.0. The cells were sonicated (10 µm amplitude, 5×30 s pulse with 1 min cooling interval) and the suspension was centrifuged at 25 000 ***g*** for 1 h at 4 °C. The supernatant was loaded into a pre-equilibrated Ni-NTA 5 ml Protino column (Macherey-Nagel) connected to an AKTA Prime Plus liquid chromatography system. Following washing with lysis buffer, the protein was eluted with an equivalent buffer containing 250 mM imidazole. The eluted fractions were concentrated on an Amicon 10 kDa centrifugal filter by washing five times with buffer containing 20 mM Tris/HCl, 100 mM NaCl and 10 % glycerol (pH 8.0), and the resulting protein was aliquoted and stored at −20 °C. The purity of the eluted protein was checked by SDS-PAGE, and its approximate concentration (~70 mg l^−1^) was calculated from absorbance measurements in a microvolume spectrophotometer (Biotek Synergy HT, ɛ=46 075 M^−1^ cm^−1^ at 280 nm).

**Table 1.** Primers used in this study

Primer name	Sequence (5′→3′)
27F	AGAGTTTGATCCTGGCTCAG
1525R	AAGGAGGTGATCCAGCC
MlrpfF	TAGAAATAATTTTGTTTAACTTTAAGAAGGAGATATACATATGGACACCATGACTCTCTTC
MlrpfR	TAGCAGCCGGATCTCAGTGGTGGTGGTGGTGGTGCTCGAGGGCCTGCGGCAGGACGAGCTC
pET21bF	CTCGAGCACCACCACCAC
pET21bR	CATATGTATATCTCCTTCTTAAAGTTAAACAAAATTATTTCTAGAG
T7promF	TAATACGACTCACTATAGGG
T7termR	GCTAGTTATTGCTCAGCGG
MiSeqBV3F	TCGTCGGCAGCGTCAGATGTGTATAAGAGACAGCCTACGGGNGGCWGCAG
MiSeqBV4R	GTCTCGTGGGCTCGGAGATGTGTATAAGAGACAGGACTACHVGGGTATCTAATCC

### Cultivation and colony forming units

A stock solution of 1,6-anhydro-MurNAc was prepared at 100 mg ml^−1^ concentration in sterile water. Reasoner’s 2A agar medium (R2A) was autoclaved and, while still hot (55 °C), supplemented with nystatin at a final concentration of 100 mg l^−1^. Immediately after the antibiotic, either the Rpf protein or 1,6-anhydro-MurNAc were added at final concentrations of 70 µg l^−1^ and 5 mg l^−1^, respectively. The media were poured into 150 mm diameter Petri dishes. Control plates containing R2A agar without Rpf protein or 1,6-anhydro-MurNAc were also prepared. The cell suspensions from the gut of the cockroaches and the soils were further diluted to 10^−2^ and 10^−3^ respectively with 50 % (v/v) sterile glycerol, and 100 µl of each dilution was spread onto the freshly prepared R2A plates, and incubated at 28 °C for 10 days. Every day, the number of colony-forming units (c.f.u.) was counted by visual inspection.

### Culture and isolation of bacterial strains G173LV and G177LV

Guzman Actinomycetes Isolation Agar (GAIA) medium was prepared by suspending in ultrapure water the following components (per litre): 4 g chitin, 2 g NH_4_Cl, 1.5 g l-arginine, 0.8 g sodium pyruvate, 0.25 g l-methionine, 0.2 g d-cysteine and 15 g agar, with pH adjusted to 8.8 before pouring. The medium was supplemented by adding a final concentration of 0.1 % (v/v) from a vitamin solution prepared by dissolving 4.6 g of Vanderzant vitamin mixture (Sigma) in 35 ml water followed by filter sterilization. Trace metals were also included by adding a solution at a final concentration of 0.2 % (v/v), which had been prepared by dissolving 200 mg FeCl_3_·6H_2_O, 40 mg ZnSO_4_·7H_2_O, 10 mg CuSO_4_·7H_2_O, 10 mg MnCl_2_·4H_2_O and 10 mg Na_2_MoO_4_ in 100 ml water and autoclaving. Finally, nystatin at 100 mg l^−1^ and recombinant *Micrococcus luteus* Rpf protein was added at a final concentration of ~50 µg l^−1^. Following inoculation of the 10^−2^ dilution from the cockroach LV, and incubation at 28 °C, the colonies G173LV and G177LV were picked and cultured onto 33 % CASO agar. The isolates were found to be pure by observation under a stereoscope and confirmed by sequencing the 16S rRNA gene using standard eubacterial primers 27F and 1525R [26]. PCR products were purified and Sanger sequenced, and the resulting almost-complete 16S rRNA gene sequences were uploaded into GenBank (Table 2). The strains were deposited at the German Collection of Microorganisms and Cell Cultures (DSMZ) and the Czech Collection of Microorganisms (CCM).

**Table 2.** Two bacterial strains isolated from the gut of the cockroach *Lucihormetica verrucosa* are probably taxonomic novelties

Strain	Genus	Family (Order)	Deposition in strain collections	16S rRNA GenBank accession	Closest type species (% 16S rRNA gene sequence similarity)
G173LV	* Streptomyces *	* Streptomycetaceae* (*Kitasatosporales*)	DSM 112694, CCM 9166	OP753711	* Streptomyces termitum* NBRC 13087^T^ (99.71%)
G177LV	* Gordonia *	* Gordoniaceae * (* Mycobacteriales *)	DSM 112678, CCM 9158	MZ182279	* Gordonia humi* CC-12301^T^ (98.95%)

### DNA extraction, amplicon sequencing and quantitative PCR

After 10 days of incubation, the agar containing the cells was transferred to 50 ml centrifuge tubes and heated at 80 °C for 15 min to melt. The tubes were centrifuged at 2 432 ***g*** for 10 min, the supernatant was discarded, and the resulting pellet was transferred to DNA extraction tubes containing ceramic beads from the NucleoSpin soil DNA isolation kit (Macherey-Nagel). DNA was purified following the instructions with the kit, with SL1 lysis buffer and enhancer SX, using a FastPrep-24cell (MP Biomedicals) bead-beater at 5 m s^−1^ applied four times at 30 s. DNA was eluted with 30 µl of SE buffer. The 16S rRNA gene hypervariable region V3–V4 was amplified using primers 341F/785R [27] with Illumina adapters (Table 1). PCR was carried in a 50 µl volume using Phusion High-Fidelity polymerase (NEB) with a temperature programme consisting of 98 °C for 1 min, 25 cycles at 98, 60 and 72 °C for 45, 45 and 30 s respectively, and a final extension at 72 °C for 5 min. The PCR product (~460 bp) was purified using a GeneRead Size Selection kit (Qiagen) and the amplicons were sequenced on an lllumina MiSeq device to produce 2×300 bp paired-end reads. Raw reads are available at the NCBI Sequence Read Archive (BioProject ID: PRJNA968073). The DADA2 [28] and Phyloseq [29] R packages were used for processing the amplicon sequencing reads and the Silva 138 Small Subunit database was used for taxonomic classification. We used the primers MiSeqBV3F and MiSeqBV4R and Sso Advanced Universal SYBR Green (Bio-Rad) supermix for the quantitative PCR (qPCR) experiment on a Real-Time StepOnePlus (Applied Biosystems) device. We used the same PCR programme as detailed for amplicon sequencing, but the number of cycles was adjusted to 35. A qPCR calibration curve was constructed using eight tenfold dilutions of *Micrococcus luteus* DSM 20030^T^, whose DNA was extracted by heating the cells at 98 °C for 10 min in the presence of 0.2 % SDS and 10 mM EDTA, and diluted tenfold prior to setting up the PCR.

## Results

### Colony-forming units

**Fig. 1.**
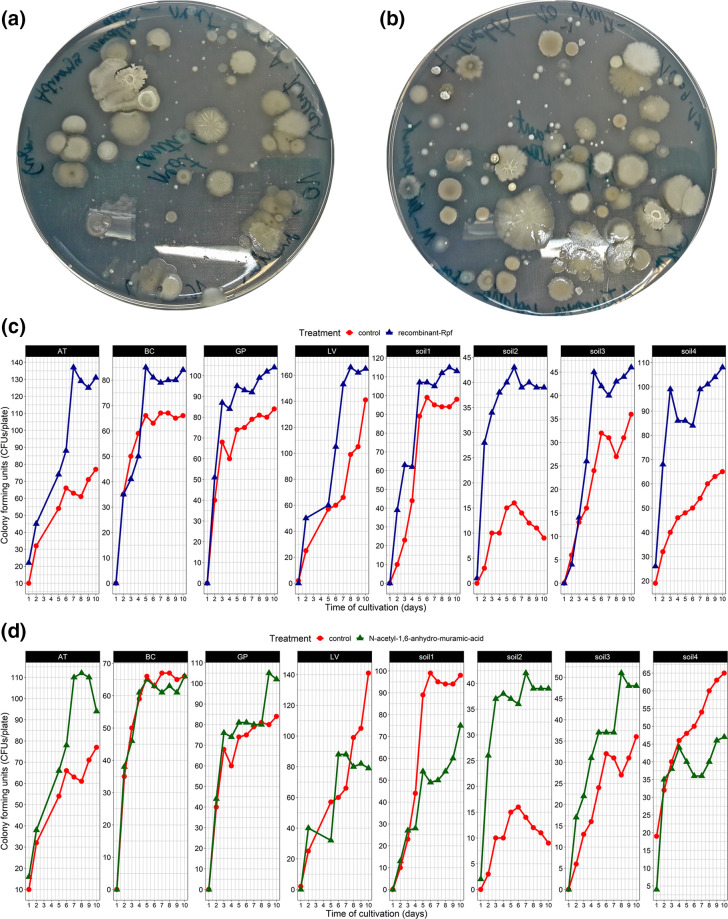
Effect of adding recombinant Rpf protein (70 µg l^–1^) or 1,6-anhydro MurNAc (5 mg l^−1^) on R2A cultivation of eight environmental samples. (a) R2A cultivation plate without Rpf addition after incubation for 10 days. (b) R2A cultivation plate with recombinant Rpf protein after incubation for 10 days. (c) Colony-forming units counted per plate for each of the eight samples with and without Rpf addition. (d) Colony-forming units counted per plate for each of the eight samples with and without addition of 1,6-anhydro-MurNAc.

Rpf addition to cultivation media markedly increased the number of colony-forming units growing on the surface of the R2A agar medium. At a concentration of 0.07 mg l^−1^, Rpf protein increased the number of colonies growing on the agar surface compared to the control (Fig. 1a, b). The number of c.f.u. after 10 days of incubation increased compared to the control in all samples (Fig. 1c). For the samples cockroach-AT and soil2, the number of colonies doubled or quadrupled when Rpf was added. The effect was much less pronounced when using 1,6-anhydro-MurNAc as a cultivation enhancer at 5 mg l^−1^ (Fig. 1d). Addition of 1,6-anhydro-MurNAc resulted in comparable c.f.u. numbers as when Rpf was added only in samples cockroach-AT and soil2; however, in the other samples no increase in the number of c.f.u. was observed.

**Fig. 2.**
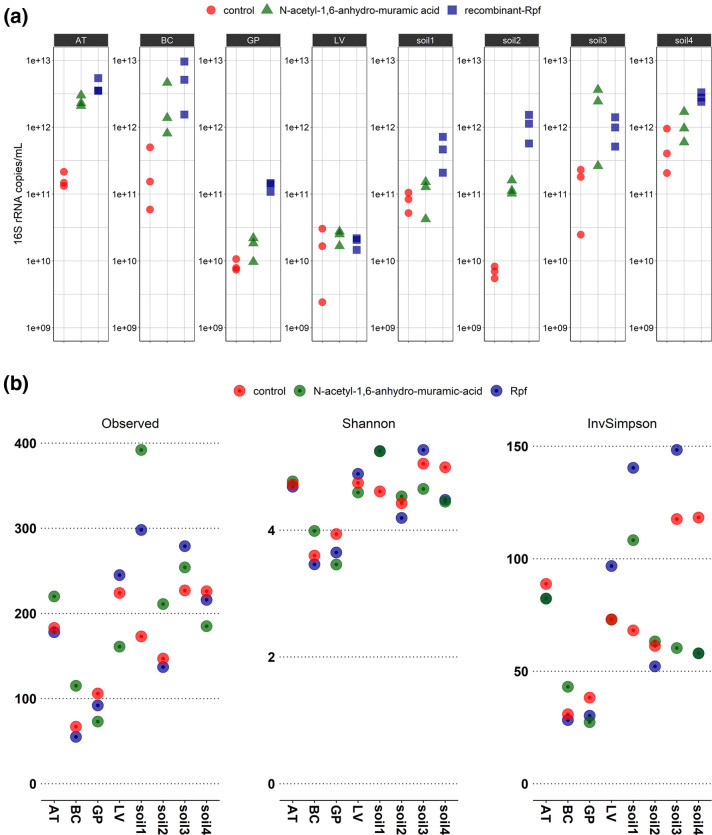
16S rRNA copy number, observed species richness and diversity indexes. (a) Copy number of the 16S rRNA gene measured by qPCR for each of the eight cultured samples under the three treatments: control, recombinant Rpf or 1,6-anhydro-MurNAc. (b) Observed species richness (number of amplicon sequence variants) and Shannon and inverted Simpson diversity indexes for the eight samples under different cultivation treatments.

The abundance of bacteria cultured for each treatment (Rpf, 1,6-anhydro-MurNAc and control) was estimated by measuring the number of 16S rRNA gene copies by using primers MiSeqBV3F and MiSeqBV4R, which showed an amplification efficiency of 2.27. Bacterial abundance ranged between 6×10^8^ and 4×10^11^ copies in control samples, whereas it was consistently higher (ranging between 1×10^11^ and 4×10^12^ copies) in cultured media supplemented with Rpf protein compared to the control (Fig. 2a). The range of increment was one or two logarithmic units, equivalent to 10–100 times more gene copies. We also observed an increment of the 16S rRNA gene copies when 1,6-anhydro-MurNAc was added to the medium. However, this effect was less pronounced compared to the increase observed when adding recombinant Rpf (Fig. 2a).

### G173LV and G177LV

Analysis of the 16S rRNA gene sequences for isolates G173LV (accession OP753711) and G177LV (accession MZ182279) revealed that they belong respectively to the genera *Streptomyces* and *Gordonia *. *Streptomyces* sp. G173LV showed high similarity to the type strain *Streptomyces termitum* NBRC 13087^T^ (identity 99.71 %), and was more distant from other closely related *Streptomyces* species such as *Streptomyces laurentii* ATCC 31255^T^ (98.57 %) and *Streptomyces roseofulvus* NBRC 13194^T^ (98.49 %). The *Gordonia* isolate G177LV was related to a number of *Gordonia* type strains, including *Gordonia humi* CC-12301^T^ (98.95 %), *Gordonia didemni* B204^T^ (98.25 %) and *Gordonia malaquae* NBRC 108250^T^ (98.04 %).

### Amplicon sequencing

To examine the effect of adding Rpf and 1,6-anhydro-MurNAc on the diversity of the cultured bacterial species, we amplified a region of the 16S rRNA gene and sequenced the amplicons using Illumina technology. The observed species richness was measured as the total unique amplicon sequence variants (ASVs) detected for each sample and treatment (Fig. 2b). Cultivation of BC and GP samples resulted in lower species richness (~100 ASVs), compared to the other two cockroaches, AT and LV (~200 ASVs detected) across the three treatments. The soil samples were comparatively richer than the cockroach samples, with soil1 yielding 400 ASVs when 1,6-anhydro-MurNAc was added to the cultivation medium. Species richness was higher in the samples cockroach LV, soil1 and soil3, cultured with Rpf protein compared to the control (Fig. 2b). Cultures with 1,6-anhydro-MurNAc showed higher species richness in cockroaches AT, BC, soil1, soil2, soil3 and soil4, compared to the control. The Shannon and inverted Simpson indexes (Fig. 2b) showed that cultures from soil1 and soil3 are the most diverse, particularly when grown in the presence of recombinant Rpf.

**Fig. 3.**
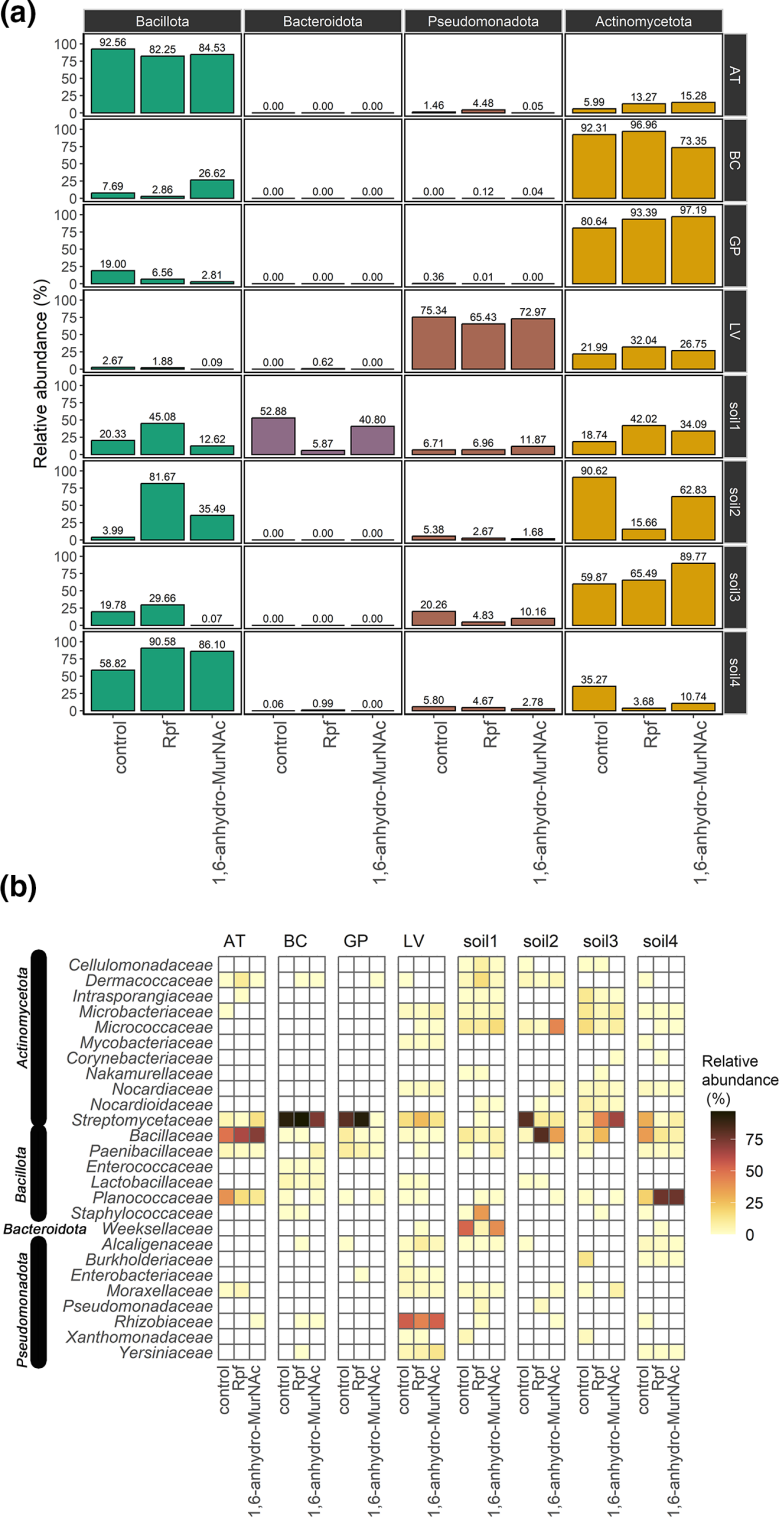
Amplicon sequencing results of eight environmental samples cultured in R2A media supplemented with recombinant Rpf protein and 1,6-anhydro-MurNAc compared with control. (a) Relative abundance of the phyla *Actinomycetota *, *Bacillota *, *Bacteroidota* and *Pseudomonadota* for the eight samples under different cultivation treatments. (b) Relative abundance at the family level for the same samples.

Taxonomic classification of each detected sequence using DADA2 allowed us to build plots of relative abundance for particular taxonomic groups. The cockroach sample AT was dominated (~90 %) by the phylum *Bacillota* (Fig. 3a), and the addition of Rpf or 1,6-anhydro-MurNAc did not increase its relative abundance. However, a minor group (~5 %) belonging to the phylum *Actinomycetota* increased in abundance when recombinant protein or 1,6-anhydro-MurNAc was added to the cultivation media. Examination of the ASVs revealed that the *Actinomycetota* that increased in abundance in AT samples were predominantly members of the families *Dermacoccaceae *, *Intrasporangiaceae* and *Streptomycetaceae* (Fig. 3b). We observed equivalent results for the three cockroaches. The major phylum in BC and GP was *Actinomycetota *, and Rpf addition increased phylum abundance from ~92 % and ~80 % to ~96 % and ~93 % respectively (Fig. 3a). The increment in relative abundance in these samples was caused exclusively by an increase in *Intrasporangiaceae* and *Streptomycetaceae* (Fig. 3b). On the other hand, in LV, the major phylum was *Pseudomonadota* (~75 %), and addition of cultivation enhancers increased the relative abundance of *Actinomycetota* from ~22 % to ~32 % with Rpf and to ~27 % with 1,6-anhydro-MurNAc, driven by the families *Nocardiaceae* and *Micrococcaceae *.

The addition of Rpf drastically increased the relative abundance of *Bacillota* in all soils, and in soil2 it increased from ~4 % to ~82 %, at the expense of the relative abundance of *Actinomycetota *. We also observed a similar behaviour in soil4. A closer examination of the *Bacillota* clades promoted in soil2 revealed that these pertained to the family *Bacillaceae *; however, in soil1 and soil4 members of the families *Staphylococcaceae* and *Planococcaceae* increased (Fig. 3b). The composition of soil1 was unique in that it contained non-negligible abundances from the four phyla, and the addition of Rpf promoted the phyla *Bacillota* and *Actinomycetota* at the quantitative expense of *Bacteroidota* (Fig. 3a). In soil3, the addition of Rpf also increased the relative abundances of both *Bacillota* and *Actinomycetota *, from ~20 % to ~30 %, and from ~60 % to ~65 % respectively.

## Discussion

Our cultivation experiments measured the c.f.u. for the same set of samples cultured under identical conditions, the only difference being the addition of either the recombinant Rpf protein or 1,6-anhydro-MurNAc. The results unequivocally showed that the addition of Rpf increases the c.f.u. across all samples. This effect had been previously reported for axenic cultivation of *Micrococcus luteus* [30], *Mycobacterium tuberculosis* and *Rhodococcus rhodochrous* [6]. Recently, it was extended to environmental samples, which revealed a remarkably diverse spectrum of resuscitated species [22, 31, 32]. The experiments presented here indicate that 1,6-anhydro-MurNAc exhibits a limited resuscitation activity on its own. However, it was less potent than the Rpf protein in increasing c.f.u. numbers. Together, our results show that additional fragments may be released during Rpf action on the peptidoglycan, and these may be responsible for the observed effect [13]. Alternatively, the limited effect of 1,4-anhydro-MurNAc on c.f.u. numbers could be caused by the fact that its concentration was not high enough or not optimal to observe the same effect as when adding Rpf.

To indirectly assess bacterial abundance, the number of 16S rRNA gene copies was determined. It confirmed the c.f.u. numbers counted on the plate, as a higher number of gene copies were detected in the samples that showed a major increment in c.f.u. The most potent effect was observed following recombinant Rpf addition on samples AT and soil2. We performed systematic literature searches in different databases and could not find an equivalent experiment; however, two studies examined the effect of adding Rpf to the soil, in contrast to the cultivation media [33, 34]. One study showed that 16S rRNA gene copies increased after adding the protein compared to the control [34]. Surprisingly the second study found that the number of bacterial 16S rRNA of copies decreased while fungal ITS copies increased [33].

*Streptomyces* sp. G173LV, isolated from the gut of the cockroach LV, was found to be closely related to the type strain of *Streptomyces termitum *, which had been isolated in 1951 from the bodies of termites [35]. This result was interesting, as it probably indicates that a clade of *Streptomyces* species have been co-evolving with the insect order Blattodea. The diversity and biological activity of *Streptomyces* spp. found in insects reveals that those associated with cockroaches and termites tend to display consistent antimicrobial activity [36, 37]. This association of highly specialized antibiotic-producing *Streptomyces* clades with cockroaches should be studied in follow-up investigations. The sequence of the almost-complete 16S rRNA gene of *Streptomyces* sp. strain G173LV exhibits high similarity (>99.7 %) to that of *S. termitum* NBRC 13087^T^, suggesting that G173LV belongs to this species. However, other *S. termitum* strains [38] have previously shown 16S rRNA similarity of >99.9 % to the type strain of *S. termitum *. Thus, a comprehensive study including the whole genome of G173LV is needed to ascertain its exact taxonomic position.

The 16S rRNA gene sequence of *Gordonia* sp. strain G177LV was <99 % identical to the most closest species *Gordonia humi *, *G. didemni* and *G. malaquae,* respectively. According to the List of Prokaryotic names with Standing in Nomenclature (LPSN), the type strains of these three species have been isolated from soil, from a marine ascidium and from sludge of a wastewater treatment plant, respectively [39]. The range of 16S rRNA gene sequence similarity values is comparable to those found for distinct species of the genus [40]. Consequently, it is suggested that G177LV may represent a novel species of the genus *Gordonia *. A detailed examination of phenotypic and genomic characters is presently under way.

Finally, we carried out sequencing experiments to examine the diversity of bacteria growing on the plates, following addition of Rpf or 6-anhydro-MurNAc to the R2A cultivation medium. Our experiment suffers from the statistical limitation that only one sequencing run was carried from every pooled sample, as we aimed to explore the effect of Rpf or 6-anhydro-MurNAc addition to the diversity of cultured taxa across as many samples as possible. The addition of 1,6-anhydro-MurNAc increased the observed ASV richness in comparison with the addition of the recombinant Rpf protein. This result was intriguing as both the c.f.u. and the 16S rRNA copy number were higher in the Rpf-treated group. We hypothesize that 1,6-anhydro-MurNAc could act as a nutritive factor, being metabolized to peptidoglycan by certain species such as *Tannerella forsythia *, which is unable to make its own *N*-acetylmuramic acid [41].

The addition of Rpf to the cultivation media increased the relative abundance of members of the *Actinomycetota *. It has been long recognized that Rpf is able to resuscitate not only members of the same species but also related taxa within the phylum *Actinomycetota* [5]. We hypothesize that certain clades are prone to be resuscitated by the Rpf from *Micrococcus luteus *, whereas other clades may be insensitive to either the Rpf protein or the 1,6-anhydro-MurNAc. In soil2 and soil4 we observed a decrease in the relative abundance of *Actinomycetota* following Rpf addition. Instead, Rpf promoted the growth of *Bacillus* in soil2 and a decrease in the relative abundance of *Streptomycetaceae *. This result is unexpected as common sense dictates that an awakening promoter is beneficial by acting on sibling cells instead of potential competitors. On the other hand, a recent report found higher c.f.u. numbers of *Paenibacillus* and *Lysinibacillus* following Rpf addition to soil samples [22]. A second study also showed that Rpf can boost the growth of *Bacillus* from sediments of the Puyang river in China [42].

We therefore suggest that addition of Rpf or 1,6-anhydro*-*MurNAc to cultivation media is a suitable method for discovering still undescribed bacterial taxa, and particularly for culturing certain clades of the phyla *Actinomycetota* and *Bacillota *. We have cultured more than five new bacterial species using this method in addition to G173LV and G177LV (unpublished results). Recombinant Rpf protein is active at nanomolar concentrations; thus, the method is a cost-effective strategy for improving the cultivation efficiency of environmental samples. The method could also be used for resuscitating old cultures, or when it becomes necessary to wake up dormant bacteria in specific experimental settings.
